# Injury Severity of Motorcycle Riders Involved in Traffic Crashes in Hunan, China: A Mixed Ordered Logit Approach

**DOI:** 10.3390/ijerph13070714

**Published:** 2016-07-14

**Authors:** Fangrong Chang, Maosheng Li, Pengpeng Xu, Hanchu Zhou, Md. Mazharul Haque, Helai Huang

**Affiliations:** 1 School of Traffic and Transportation Engineering, Central South University, Changsha 410075, China; fangrongchang@csu.edu.cn (F.C.); maosheng.li@csu.edu.cn (M.L.); 144212053@csu.edu.cn (H.Z.); 2 Department of Civil Engineering, The University of Hong Kong, Pokfulam Road, Hong Kong 999077, China; xh150723@163.com; 3 Centre for Accident Research and Road Safety—Queensland (CARRS-Q), Civil Engineering and the Built Environment, Science and Engineering Faculty, Queensland University of Technology, Brisbane 4059, Australia; m1.haque@qut.edu.au

**Keywords:** injury severity, motorcyclist, unobserved heterogeneity, mixed ordered logit, China

## Abstract

Issues related to motorcycle safety in China have not received enough research attention. As such, the causal relationship between injury outcomes of motorcycle crashes and potential risk factors remains unknown. This study intended to investigate the injury risk of motorcyclists involved in road traffic crashes in China. To account for the ordinal nature of response outcomes and unobserved heterogeneity, a mixed ordered logit model was employed. Given that the crash occurrence process is different between intersections and non-intersections, separate models were developed for these locations to independently estimate the impacts of various contributing factors on motorcycle riders’ injury severity. The analysis was based on the police-reported crash dataset obtained from the Traffic Administration Bureau of Hunan Provincial Public Security Ministry. Factors associated with a substantially higher probability of fatalities and severe injuries included motorcycle riders older than 60 years, the absence of helmets, motorcycle riders identified to be equal duty, and when a motorcycle collided with a heavy vehicle during the night time without lighting. Crashes occurred along county roads with curve and slope alignment or at regions with higher GDP were associated with an elevated risk of fatality of motorcycle riders, while unsignalized intersections were related to less severe injuries. Findings of this study are beneficial in forming several targeted countermeasures for motorcycle safety in China, including designing roads with appropriate road delineation and street lighting, strict enforcement for speeding and red light violations, promoting helmet usage, and improving the conspicuity of motorcyclists.

## 1. Introduction

Motorcycle crashes are one of the leading causes of unnatural death worldwide. Almost half of all deaths on the world’s roads are among those with the least protection: motorcyclists (23%), pedestrians (22%) and cyclists (4%) [1]. In particular, motorcyclists accounted for 9% of road fatalities in Europe, 20% in America, and 34% in both western Pacific and southeast Asia countries [1]. As the most vulnerable road user, motorcycle riders are susceptible to being fatally injured if involved in a collision [2]. After controlling for per vehicle mile traveled, motorcyclists were reported to suffer a 26-fold higher risk of death in a crash than those driving other types of motor vehicles [3].

Motorcyclist injuries continue to be a considerable public health concern in China. The total number of motorcycles registered in China increased overwhelmingly from 2.5 million (23% of all registered motorized vehicles) in 1987 to 49.9 million (nearly 65%) in 2002, reaching 91.5 million units (about 35%) in 2014 [4]. In accordance with this growth, the number of fatalities due to motorcycle crashes also raised by about 6.7 fold, from 3078 in 1987 to 20,773 in 2002. Although this figure gradually declined afterwards (reducing to 10,411 in 2014), the proportion of traffic fatalities sustained by motorcyclists is still relatively high, i.e., approximately 20% [4]. The situation appears to be more serious in southern China where motorcycles are prevalently used. For instance, motorcyclists represent more than 24% of total traffic fatalities in Hunan Province in 2014.

Despite the pressing safety challenge brought by motorcycles, a limited number of studies have specifically focused on China’s motorcyclist-related safety issues [5,6,7,8,9]. Existing research concerning motorcyclist injuries are mostly derived from the developed regions [10,11,12,13,14,15,16,17,18,19,20,21,22,23,24,25]. The causal relationship between injury outcomes of motorcycle crashes and potential risk factors in China remains unknown.

In terms of motorcycle styles, usage and trip purpose, there is a substantial difference between most developed countries and China. Motorcycles with larger engine capacities are preferred by single riders for occasional touring and leisure purposes in the US, Canada, and Europe [2]. However, in China, due to local governors’ concern about motorcycle-related issues including traffic casualties, traffic chaos, and pollution, motorcycles are prohibited on public roads in many large cities such as Beijing, Shanghai, and Guangzhou. As a convenient and affordable means of transportation, motorcycles with lower capacities are extensively used by low- and middle-income families for daily commuting in less developed cities and rural areas in China. In addition, due to the distinction in cultural foundations, traffic policies, road environments and driver characteristics [26], the risk factors contributing to the severity of motorcyclist injuries in China are expected to be different from those in developed countries. On one hand, lessons learnt from developed countries can be of great value to China as almost all developed and motorized countries have experienced stages of motorcyclists’ high fatality. On the other hand, China has unique features that require deliberate assessment of the applicability of a variety of countermeasures.

The present study intends to investigate the injury risk of motorcycle riders involved in motor vehicle crashes in China. In this study, a motorcycle rider is the operator only, and a passenger refers to any person seated on the motorcycle but not in control of the motorcycle. In any combined reference, they are represented as the motorcyclists. Based on a comprehensive dataset collected from Hunan Province of China, a mixed ordered logit model is established to estimate the likelihood of fatal and severe injuries of motorcycle riders as a function of various factors, such as the detailed motorcyclist characteristics, crash characteristics, geometric design and environmental features. The findings could be used for the evidence-based interventions for policy-making decisions to reverse the adverse motorcycle crash trend and to mitigate motorcyclist injuries in China.

## 2. Literature Review

Motorcycle-related crashes have long been a major research concern. Researchers have attempted to establish predictive models to investigate the determinants of motorcyclist injuries in traffic crashes in US [17,21,24,25], Canada [22], UK [14,15,16], France [13], Australia [20], Singapore [11], Korea [23], and Taiwan [12].

A wide variety of factors have been explored, including the demographic attributes of motorcyclists, traffic characteristics, road geometry and environmental features. Generally, the following factors were reported to significantly increase the injury severity sustained by motorcyclists: increased age [13,14,15,16,17,21], riding without helmets [17,21,24,25], alcohol use [17,21,22,23,25], speeding [12,15,17,25], motorcycle with a larger engine size [11,13,14,16], collisions with stationary objects or heavier vehicles [12,17,21,25], and motorcyclists at fault [11,17]. Conflicting findings were reported when it comes to the effects of motorcyclist gender and lighting condition. For example, female motorcycle riders were found more likely to be involved in serious injuries in most previous studies [13,17,21,22], whereas Shaheed et al. [24] drew an opposite conclusion. Meanwhile, Schneider and Savolainen [21] indicated the complete darkness associated with a lower likelihood of fatality owing to the risk compensation (i.e., motorcycle drivers in darkness tend to adjust their behavior by decreasing riding speeds, which has a positive effect on injury severity), while the absence of light was suggested related to a higher level of injury in most other cases [12,13,17,23,24].

Various methods, such as on-site investigation, mathematical modeling, and simulation, have been used to evaluate the severity of motorcyclist injuries. Of these, econometric modeling approaches, which focus on the analysis of injury severity from the perspective of overall safety and its econometric implications, hold considerable promise. Conditional on a crash having occurred, econometric crash-severity models cover a broad range of methods, including the binary logit models [16], ordered logit/probit models [11,14,15,22,23], generalized ordered logit models [22], multinomial logit models [10,17], nested logit models [17], proportional odds models [12], a latent class multinomial logit model [25], and mixed (random parameters) logit models [24,27].

Since the injury severities are inherently ordered, the ordered–discrete choice model is a superior alternative to account for the ordinal nature of response outcomes [11,14,15,28,29]. One limitation imposed by the traditional discrete choice approach (including the standard multinomial and ordered logit models) when applied to the crash-injury data is that it cannot allow for cross-individual heterogeneity. In reality, as each individual being analyzed has specific characteristics that may influence the severity outcomes differentially, the impacts of explanatory variables are expected to vary across observation. Another concern is that some of the factors that influence the severity of crashes are not observed or are nearly impossible to collect. If these unobserved factors (i.e., often referred to as unobserved heterogeneity [30] are correlated with the observed ones, biased parameters will be estimated and incorrect inference could be drawn [30,31]. Recent studies [32,33] have therefore introduced the mixed ordered response model to address the unobserved heterogeneity issue and the potential variations in the effects of contributing factors.

Based on all of the above, to meet the urgent need to improve motorcyclist safety in China, the mixed ordered logit model was adopted to achieve an explicit understanding of factors influencing the injury levels of motorcycle riders in motor vehicle crashes, with which effective countermeasures could be scientifically proposed.

## 3. Data Collection

The crash data was obtained from the Traffic Management Sector-Specific Incident Case Data Report, which was maintained by the Traffic Administration Bureau of Hunan Provincial Public Security Ministry. Hunan is located in South Central China with an area of 211,800 km^2^ and a population of 67.4 million. It is composed of 14 prefectures, 122 counties and 2576 townships. The province ranked 10th among 31 provinces in terms of Gross Domestic Product (GDP) value of 2.71 trillion CNY (i.e., 440.29 billion USD) in 2014 [34]. The number of registered motorcycle in Hunan was about 5 million in 2014, accounting for 52% of all motorized vehicles [4].

A total of 20,027 motorcycle crashes was reported in Hunan in 2014. Of these, about 81% were motorcycle–motor vehicle crashes. In comparison, single motorcycle crashes and motorcycles colliding with non-motor vehicle crashes, respectively, represented 6% and 13%. Given that motorcyclists involved in motor-vehicle crashes tended to sustain severe casualties (e.g., in our dataset, 88% of fatalities in motorcyclists were caused by the collisions between motorcycles and motor vehicles), we particularly focused on this crash type. Other crash types, such as the single motorcycle crashes and motorcycles colliding with non-motor vehicles were explicitly excluded. Due to the incomplete information in police records, 5316 crashes were retrieved for further investigation.

In China, the injury severities are commonly assessed by police officers at the scene of crash and categorized as property damage only (i.e., no injury), slight injury (i.e., non-disability injury), serious injury (i.e., disability injury) and fatality (i.e., immediate or subsequent death from injuries within seven days after a crash). By aggregating the crash and casualty injury profiles, the predictor variables reflecting the demographic characteristics of motorcyclists (i.e., age and gender), crash characteristics (i.e., helmet usage, passenger on board or not, duty judgment, motorcycle type, collision vehicle, and collision type), geometric design features (segment or intersection, signal type, road classification and alignment), together with the environment factors (i.e., crash time, weather, and lighting condition) were collectively extracted. The GDP of corresponding counties was also collected to reflect the economic development levels [35].

Given that some of the factors affecting crash risk are substantively different at intersection and non-intersection locations [21,36,37], separate models were developed to independently estimate the effects of related factors on the injury severity of motorcycle riders resulting from crashes at intersection and non-intersection locations. Table 1 illustrates the variables available for model development, with the proportions of the categorical variables above and the descriptive statistics of the continuous variables below. Interestingly, more than 90% of crash-involved motorcycle riders were male and the prevalence rate of helmet use in our sample was fairly low, i.e., 28.2% and 30.2% in non-intersection and intersection crashes, respectively.

## 4. Methodology

The multiple ordinal categories for injury severities (i.e., j=1,2,3,4) facilitated the application of ordered logit model:

(1) $$y_{i}^{*}=\beta^{\prime }X_{i}+\varepsilon_{i}$$

where $y_{i}^{*}$ represented the latent injury risk propensity for motorcycle rider i(i=1,2,…,n); $X_{i}$ was an (L+1)×1 column vector of explanatory variables (including the constant term); **β** was the corresponding vector of regression coefficients to be estimated (i.e., $\left[\beta_{1},\beta_{2},\ldots ,\beta_{p}\right]$); and *ε_i_* denoted the random error term assumed to be identically and independently standard logistic distributed.

The latent propensity $y_{i}^{*}$ was mapped to the observed injury severity level $y_{i}$ through the threshold $\mu_{j}$ ($\mu_{0}=-\infty$ and $\mu_{4}=\infty$)

(2) $$y_{i}=\left\{ \begin{array}{llll} 1 & \text{if} & -\infty \leq y_{i}^{*}\leq \mu_{1} & \left(\text{property damage only}\right)\\ 2 & \text{if} & \mu_{1}\leq y_{i}^{*}\leq \mu_{2} & \left(\text{slight injury}\right)\\ 3 & \text{if} & \mu_{2}\leq y_{i}^{*}\leq \mu_{3} & \left(\text{serious injury}\right)\\ 4 & \text{if} & \mu_{3}\leq y_{i}^{*}\leq \infty & \left(\text{fatality}\right) \end{array}\right.$$

where $\mu_{1}$, $\mu_{2}$, and $\mu_{3}$ were threshold values to be estimated together with the **β**.

We then had the following predicted probabilities:

(3) $$\begin{array}{l} \text{Prob}\left(y_{i}=1|X_{i}\right)=F\left({\hat{\mu }}_{1}-X_{i}\hat{\beta }\right)\\ \text{Prob}\left(y_{i}=2|X_{i}\right)=F\left({\hat{\mu }}_{2}-X_{i}\hat{\beta }\right)-F\left({\hat{\mu }}_{1}-X_{i}\hat{\beta }\right)\\ \text{Prob}\left(y_{i}=3|X_{i}\right)=F({\hat{\mu }}_{3}-X_{i}\hat{\beta })-F({\hat{\mu }}_{2}-X_{i}\hat{\beta })\\ \text{Prob}\left(y_{i}=4|X_{i}\right)=1-F({\hat{\mu }}_{3}-X_{i}\hat{\beta }) \end{array}$$

where *F*(•) was the cumulative distribution function with the mathematical form:

(4) $$F(z)=\frac{\exp(z)}{1+\exp(z)}$$

One restriction of above basic ordered logit model is that it holds the regression coefficients to be fixed across individual observations. To this end, the mixed ordered logit model was proposed, assuming the coefficients to be randomly distributed with possibly heterogeneous parameters generated by [38]:

(5) $$\beta_{ik}=\beta_{k}+\varphi_{ik}$$

in which $\varphi_{ik}$ was a randomly distributed term (e.g., a normally distributed term with a mean of zero and variance $\sigma_{k}^{2}$). In practice, a random parameter $\beta_{ik}$ is used whenever ${\hat{\sigma }}_{k}$ is significantly greater than zero; otherwise, the parameter is fixed across the individuals.

Now, the conditional probability entering the log likelihood function became:

(6) $$\text{Prob}\left[y_{i}=j|X_{i},\beta_{i}\right]=F(j,\mu ,\beta_{i}^{\prime }X_{i})$$

The estimation of the above mixed ordered logit model could be based on the simulated maximum likelihood approach with 200 Halton draws (see Train [39] for detailed explanation of simulated maximum approach and Halton draws). For the functional form of the parameter density function, consideration was given to normal, lognormal, uniform, and triangular distributions.

For model comparison and selection, the Akaike Information Criterion (AIC) was used. AIC simultaneously considers the goodness-of-fit and complexity of model, which is defined as:

(7) AIC=−2ln(L)+2K

where *L* is the maximum value of the likelihood function for the model, and *K* is the number of estimated parameters in the model.

## 5. Results and Discussion

For model specification, a correlation test using the variance inflation factor (VIF) was conducted to ensure the non-existence of any highly correlated variables. Results revealed that the maximum value of VIF was 3.7 between signal control and lighting configuration, implying that no strong multi-collinearity among independent variables existed in our dataset. The likelihood ratio test was then used to guarantee that each added variable significantly improved the overall model performance.

For the purpose of comparison, the basic ordered logit model was also estimated. As such, four models with two for intersections and the other two for segments were eventually developed. Performances of the calibrated models are compared in this section first, followed by the presentation and interpretation of the parameter estimates.

### 5.1. Model Comparison

Table 2 shows the results of goodness-of-fit measures for the models. Undoubtedly, incorporating the random parameters resulted in an increase in model complexity but a considerable improvement in overall fit as measured by *LL*(**β**). In particular, the mixed form to model the injury of motorcyclists at non-intersection locations was definitely superior in terms of AIC statistics (approximately 20 points lower) and likelihood-ratio test (at 1% level of significance). Although there was no substantial difference in goodness-of-fit between fixed and mixed model types when applied to intersection crash data, the presence of random parameters explicitly demonstrated the existence of heterogeneity in the effects of risk factors. Therefore, the following section presents the interpretation of significant factors obtained from the mixed ordered logit models.

### 5.2. Parameter Estimates

Table 3 summarizes the parameter estimates in mixed ordered logit models for non-intersection and intersection crashes, respectively. A 95% level of significance was used as the threshold to determine whether the parameters differed from zero. Insignificant variables were removed from the final models. Since the interpretation of coefficients are complicated and do not directly indicate the magnitude of the impacts of the variables estimated, investigating the pattern and trend of marginal effects is more intuitive and insightful [38]. Table 4 thus provides the results for marginal effects, which could be interpreted as the average percentage change in the probability due to the variation in independent variables.

As illustrated in Table 3 and Table 4, the following factors were associated with a significantly higher probability of injury in both non-intersection and intersection crashes: motorcycle riders older than 60 years, the absence of helmets, motorcycle riders identified to be equal duty, and two-wheel motorcycles collided with a heavy motor vehicle in complete darkness. Crashes occurring on county roads with curves and slope alignments or at regions with higher GDP tended to suffer an elevated risk of fatality, while unsignalized intersections without curved or sloped approaches were less likely to lead to fatal and serious injuries. For all the parameters assumed to be random, the normal distribution was revealed to provide the best statistical fit. The effects of these significant contributing factors were further discussed below.

#### 5.2.1. Motorcyclist Characteristics

The involvement of motorcyclist riders over 60 years old was found to have a significant effect on injury severities. Probabilities of fatality at non-intersections and intersections were, respectively, 55% and 44% higher for riders older than 60 years. Elder riders being usually weaker in terms of physiological condition and perception of safety and slower to react in hazardous situations are likely to be severely injured if they are involved in a crash. Similar findings have been reported earlier [13,14,17,21]. The variable also produced a normally distributed random parameter with a mean of 0.639 and a standard deviation of 1.167 in the intersection model. Given these distributional parameters, 71% of the distribution is greater than zero, suggesting that 71% of the aged motorcycle riders involved in the crashes during the analysis period had increased probability of severe injury, while the remaining were less likely to be severely injured in crashes at intersections. This result probably reflects the fact that riders within the same age group usually vary in physical abilities, driving habits and other unobserved factors [36].

The absence of helmets was also found to significantly increase the injury severity of riders, with the probability of fatality approximately 32% and 34% higher than those wearing helmets at non-intersections and intersections, respectively. The protective effect of helmet use on injury outcomes has been well documented [40]. This indicator variable also led to a significant random parameter with a mean of 0.531 (or 0.707) and a standard deviation of 0.870 (or 0.597) in the non-intersection (or intersection) model. In addition, 73% (or 88%) of the distribution was greater than zero in non-intersection (or intersection) models, implying that the majority of motorcycle riders without helmets sustained a higher probability of injury risk in both intersection and non-intersection crashes. This finding is likely to illustrate the complex interaction between helmet usage and motorcyclist riding behavior. For instance, the helmeted riders are prone to be a self-selected group with good riding habits and skills, whereas those without helmets tend to be more reckless. The above findings might be neglected under a fixed-parameter analysis, introducing a bias because of the unaccounted heterogeneity.

#### 5.2.2. Crash Characteristics

In China, the responsibilities of the parties involved in road traffic crashes are typically determined by police officers at the scene. It is usually divided into five levels as total, primary, equal, secondary and no responsibility. The total responsibility is justified when the crash is fully caused by the fault of one party. If the collision is caused by two or more parties’ faults, every party has therefore to bear certain responsibility on the basis of irregular behaviors and corresponding severity outcomes. For simplicity, those with secondary or no responsibilities are combined as the minor duty in the present study, while those with full and primary responsibilities are deemed as the major duty. Inconsistent with studies in Singapore [11,19], we found that compared with those with major duty, motorcycle riders identified to be of equal duty appeared to sustain higher probability of fatality by 21% and 45% in crashes at non-intersection and intersection sites, respectively. This contradictory result could be explained by different jurisprudence behind the liability determination of parties involved in traffic crashes. China is a country with a tradition to protect the vulnerable road users [41]. Provided that the motor vehicle driver failed to take appropriate and timely actions to avoid the collisions with motorcycles, the driver had to bear a dominating portion of the liability. Likewise, if the motorcyclist was judged to take the main responsibility, it implied that the motor-vehicle driver had noticed the irregular behaviors of motorcyclist and took corresponding measures in advance. Therefore, it is expected that injuries of motorcycle riders with major duty were less serious compared with their counterparts. In addition, the estimated parameter for this variable was found to be random with a mean of 0.294 (or 0.691) and a standard deviation of 0.882 (or 0.412) in the non-intersection (or intersection) model. Given these distributional parameters, 63% (or 95%) of motorcycle riders determined to be equal duty had a higher probability of severe injury when collided with a motor vehicle at non-intersections (or intersections).

Towards the effect of motorcycle type, the parameter for the indicator variable of three-wheel motorcycles was observed to be random and significant. Specifically, the probabilities to be fatally injured in non-intersection and intersection related crashes were, respectively, reduced by 57% and 47% when motorcyclists operated three-wheel motorcycles. This result is intuitive to some extent. Recall that the three-wheel motorcycles typically possess a larger size and are commonly used to convey goods in daily life in China. This design occupies a larger space to run, which could easily attract the visibility to other road users. In addition, the good balance and heavy mass imposed by the three-wheel motorcycles (i.e., one leading front wheel with two driven rear wheels) may act favorably in reducing the injury severity in case of a crash. In addition, the variable resulted in a normally distributed random parameter with a mean of −1.223 (or −1.241) and a standard deviation of 1.103 (or 1.440) in non-intersection (or intersection) related crashes, indicating that 87% (or 81%) of motorcyclists riding a three-wheel motorcycle were less likely to be severely injured in non-intersection (or intersection) related crashes.

Collisions with heavy vehicles, such as pickup trucks and tractor-trailers, were proved to dramatically increase the likelihood of fatality by 77% and 102% in non-intersections and intersection crashes, respectively. This was consistent with previous research [17,25]. The variable also produced a random parameter that was normally distributed with a mean of 0.909 (or 1.260) and a standard deviation of 0.683 (or 0.796) in non-intersection (or intersection) model. Given these distributional parameters, 91% (or 94%) of motorcycles colliding with heavy vehicles at non-intersections (or intersections) were more likely to result in severe injuries.

#### 5.2.3. Road Geometric Design Features

Roads in China can be categorized as the national, provincial, county, township, and accommodation roads in terms of administrative hierarchy. Typically, the national and provincial roads are also known as principal arteries. Compared to county roads, a lower risk of serious injuries for motorcycle riders was observed on national and provincial roads, with the probabilities of fatality and severe injury about 24% lower in the non-intersection model. One potential reason may be that the national and provincial roads in China generally have higher design levels of safety protection facilities and better safety performance with greater enforcement activities [42]. In contrast, due to less traffic volume and a lower degree of police presence, motorcyclists on county roads in China are more likely to engage in risky behaviors, such as non-helmet wearing, excessive speeding, and illegal overtaking. For example, riders on county roads were reported to wear helmets less despite the legislation requiring helmet use on all road types [5].

The ability to control motorcycles and the field of vision are deemed to be limited on roads with curves and slopes. Consistent with other studies [36,43], curved segments with slopes were also verified to increase the injury severity in our study. The probability of fatalities for motorcycle riders increased by 24% when collisions occurred along curved segments with slopes. Accordingly, motorcyclists driving at intersections with straight approaches were less likely to be injured fatally, with the corresponding probability about 22% lower.

Contrary to conclusions from Singapore [11] and UK [15], our empirical analysis indicated that the injury severity of motorcycle riders tended to increase at signal-controlled intersections in China. The probabilities of fatalities and severe injuries increased by about 26% when crashes occurred at intersections with signals. One potential reason is that it is common to observe motorcyclists running the red lights in China [26]. The sudden and unexpected appearance of motorcycles makes it difficult for motor vehicle drivers to yield in time. Another is that motorcyclists often accumulate at stop lines before motor vehicles during the red phase. The practice of earlier discharge during the initial green period is particularly risky for resulting in a serious collision with the across vehicles [18]. The unprotected left-turn phase has also been reported as one of the major contributing factors for the vulnerability of motorcycles at signalized intersections [44].

#### 5.2.4. Environmental Characteristics

As expected, the probability of fatalities for motorcycle riders decreased by 36% and 65% if crashes occurred in daylight at non-intersections and intersections, respectively. Additionally, compared with incomplete darkness, better lighting conditions were also beneficial to reduce the severity of intersection crashes, which lowered the likelihood of fatality and severe injury by 37% and 39%, respectively. This is expected as drivers have better visibility and more reaction time with lighting configuration [45]. Similar findings were also suggested in previous studies [12,13,17,23,24]. Meanwhile, the variable led to a significant random parameter which was normally distributed with a mean of −0.829 and a standard deviation of 0.635 in the intersection model. Given these distributional parameters, 90% of the distribution was less than zero. This result indicated that the majority of crashes (i.e., 90%) occurring at intersections with lighting configuration were less likely to lead to severe injuries.

The economic development is supposed to be closely associated with safety awareness, driving behaviors and conditions of transport facilities, thus having an indirect influence on safety outcomes. Law et al. [35] suggested that motorcycle deaths increase with increasing motorization in early stages of development. The deaths subsequently decrease when technical, policy and political institutions respond to demands for increased safety. In this study, we found that regions with higher GDP were more likely to sustain a higher likelihood of fatality, i.e., the probability of fatalities for motorcycle riders at non-intersections increased by 11% with one unit increase in GDP values. China has witnessed astounding economic growth in the last decade. This allows more households to own motor vehicles and more individuals to drive. The motorcycle injuries have become an unwanted consequence of this urbanization and motorization. Hence, evidence-based interventions specific to motorcyclist safety are badly in need. Based on the statistically significant factors identified combined with the consideration of China’s unique motorcycle-related features, several potential safety strategies are discussed and suggested in detail in the following section.

## 6. Safety Implications

Head injury is a major cause of death in motorcycle crashes. Previous studies strongly supported that wearing a helmet effectively protects motorcycle riders from the high risks of head and spinal injuries [46,47], as well as reducing the length of hospital stay and corresponding medical costs of injured riders [40]. Our empirical study also demonstrated that riders without helmets in China are associated with an elevated likelihood of fatality.

Early in 1988, “The Road Traffic Regulations of People’s Republic of China” made helmet usage mandatory for all motorcycle riders throughout the country. Despite the existing national legislation, the overall prevalence of helmet use remains low, with estimated rates ranging from 30% to 70% [5,8]. Particularly, only 37% of drivers were observed properly wearing standard helmets in a developed city in Southern China [8]. This statistic was more alarming for pillion passengers, with only 12.9% exhibited wearing a motorcycle helmet correctly [8].

The main reason motorcyclists reported wearing helmets was not to “prevent head injury” but to “cope with police” [5]. According to the “Law on Road Traffic Safety of the People’s Republic of China” enacted since May 2004, the penalty for the absence of a safety helmet is kept at 20–200 CNY (about 3–30 USD). As a contrast, the per capita disposable income has increased by more than three-fold over the past decade, raising from 6226 CNY (about 1000 USD) in 2004 to 20,167 CNY in 2014 (about 3000 USD) [34]. The immature driving group and the low-cost law violations along with the ineffectiveness of law enforcement have induced the high helmet non-usage in China.

Therefore, public education programs regarding helmet use are extensively needed to reduce motorcyclist fatalities in China. Such strategies should target all motorcyclists (including motorcycle riders and pillion passengers) and address the message that the riders are only effectively protected when a standard motorcycle helmet is properly worn. Meanwhile, to achieve a good compliance rate, the government could consider enhancing the visibility of enforcement activities and to improve the penalties for un-helmeted motorcycle riding.

Another available countermeasure is to segregate the vulnerable road users from mixed traffic by setting exclusive motorcycle lanes. Experience from Malaysia shows that the provision of exclusive lanes reduces motorcycle fatalities by 600% [48]. However, this costly measure seems inappropriate to generalize, given the prevalence of restricting motorcycles in many cities in China (According to the incomplete statistics, up to 170 cities in China have executed the motorcycle restricting rule [49]). Alternative road facilities programs, such as road alignment optimization and illumination configuration, are regarded to be feasible to enhance motorcyclist safety, since our results suggested that curved and sloped roads without lighting conditions significantly increase the injury severity (see Table 4). These remedial actions should particularly focus on county roads, as our sample illustrated that 69% of motorcycle crashes occurred on county roads (see Table 2). This road type was also proved to sustain an elevated risk of fatal and severe injuries.

Inconsistent with the studies from developed countries [11,15], intersections with signal control were more likely to lead to severe injury for motorcyclists in China. This elevated injury risk may have been modified by motorcyclists’ risk-taking behaviors, i.e., the red light violation and earlier discharge during initial green period. Thus, transportation authorities should consider providing more education programs to help motorcycle riders obey traffic rules and ride sensibly. In the meantime, more infrastructural facilities such as red light cameras, safer crossing and stay facilities should be developed for motorcyclists at signalized intersections [50]. Such direct countermeasures could improve the mobility and minimize the vulnerability of motorcycle riders in the pre-crash phase.

In addition, inexperience with riding is believed to be monotonically associated with a higher risk of motorcycle crashes and injuries [51]. Formal rider training is expected to increase riding skills and thus reduce the risk of motorcycle injuries [51]. However, controversies over the benefits of training courses arises, as riders who received training were reported to have no significant reduction in the risk of motorcycle crashes relative to those without the training [52]. In addition, no significant differences in traffic violations and costs of medicinal treatment were detected between trained and untrained motorcyclists [52]. Due to the absence of investigations concerning the effectiveness of motorcycle training in China, we intend to be conservative about this safety strategy. Future research effort is required on this topic.

Finally, other successful prevention programs, including the enforcement of speed violation and the conspicuity of motorcyclists (e.g., wearing a reflective vest while driving in dark hours), could be directly undertaken by China. These interventions are universally effective [53], and have a high benefit–cost ratio of implementation.

## 7. Conclusions

This study investigated the injury severity sustained by motorcycle riders involved in traffic crashes in China through an analysis of data from Hunan Province of China. To account for the ordinal nature of response outcomes and unobserved heterogeneity, a mixed ordered logit model was developed. The following factors were reported to be associated with a significantly higher probability of fatalities and severe injuries: motorcycle riders older than 60 years, the absence of helmets, motorcyclists identified with equal duty, and two-wheel motorcycles colliding with a heavy vehicle in darkness. Crashes occurring on county roads with curves and slope alignments or at regions with higher GDP were prone to suffer an elevated risk of fatality, while intersections with straight approaches and with no signal controls were less likely to lead to fatal and serious injuries.

Transfer of effective interventions for motorcycle injuries from developed countries to China is necessary and highly desirable. However, an understanding of the feasibility, economic cost, and potential barriers to implement these countermeasures is vital for successful transfer. China is totally different from other countries in the prevalence of motorcycle rides, purpose of motorcycle usage, amount of riding exposure, road environment, and traffic policies. If these differences are not explicitly considered, applying risk factor analytical results and safety programs from other regions, particularly to costly road engineering projects (e.g., setting exclusive lanes for motorcycles), might not be applicable. According to the significant risk factors identified in this study, a number of evidence-based safety strategies specific to China could be recommended: promotion of helmet usage, enforcement of speed and red light violations, clearer road delineation and street lighting systems, and improving the conspicuity of motorcyclists.

It should be noted that this study was based on the police-reported crash dataset. Considering the inadequacies in police reports (e.g., the possibility of under-reporting of less severe crashes and the unavailability of relevant variables), we highly advocated the integration of police-reported data and other data sources (e.g., questionnaire surveys, field observations, and driving simulations) to achieve a more explicit understanding of the casual mechanism underlying motorcycle crashes.

## Figures and Tables

**Table 1:** Descriptive statistics of variables included in the models.

Variable	Attributes		Count (Proportion)	
			Non-Intersections	Intersections
Injury severity	Fatality		536 (13.4%)	158 (12.1%)
	Serious injury		326 (8.1%)	94 (7.1%)
	Slight injury		2382 (59.5%)	857 (65.4%)
	Property damage only *		761 (19.0%)	202 (15.4%)
Age	Under 18		172 (4.3%)	53 (4.0%)
	18 to 60 *		3536 (88.3%)	1169 (89.2%)
	Above 60		297 (7.4%)	89 (6.8%)
Gender	Male		3826 (95.5%)	1223 (93.3%)
	Female *		179 (4.5%)	88 (6.7%)
Helmet usage	With helmets *		1129 (28.2%)	396 (30.2%)
	Without helmets		2876 (71.8%)	915 (69.8%)
Passenger	With passenger		1127 (28.1%)	416 (31.7%)
	Without passenger *		2878 (71.9%)	895 (68.3%)
Duty	Minor		1587 (39.6%)	540 (41.2%)
	Equal		645 (16.1%)	296 (22.6%)
	Major *		1773 (44.3%)	475 (36.2%)
Motorcycle type	Two-wheel motorcycle		3445 (86.0%)	1146 (87.4%)
	Three-wheel motorcycle *		560 (14.0%)	165 (12.6%)
Collision vehicle	Heavy vehicles		658 (16.4%)	190 (14.5%)
	Light motor vehicles *		3347 (83.6%)	1121 (85.5%)
Collision type	Moving vehicles		2649 (79.1%)	1179 (90.0%)
	Others *		698 (20.9%)	132 (10.0%)
Signal	No signal		2246 (56.1%)	541 (41.2%)
	Marked		1672 (41.7%)	647 (49.4%)
	Signal Control *		87 (2.2%)	123 (9.4%)
Road	National and provincial roads		885 (22.1%)	635 (48.4%)
classification	County roads *		3053 (76.2%)	649 (49.5%)
	Others		67 (1.7%)	27 (2.1%)
Road alignment	Straight		2245 (56.0%)	955 (72.8%)
	Curve *		1339 (33.5%)	77 (5.9%)
	Curve and slope		421 (10.5%)	279 (21.3%)
Day of week	Weekday (Monday–Friday) *		2930 (73.2%)	978 (74.6%)
	Weekend (Saturday and Sunday)		1075 (26.8%)	333 (25.4%)
Time of day	Peak time (7 a.m.–9 a.m., 5 p.m.–8 p.m.)		1334 (33.3%)	439 (33.5%)
	Non-peak time (9 a.m.–5 p.m., 8 p.m.–6 a.m.) *		2671 (66.7%)	872 (66.5%)
Weather	Rainy/Snowy/Foggy/Windy		563 (14.0%)	165 (12.6%)
	Sunny *		3442 (86.0%)	1146 (87.4%)
Light	Daylight		2849 (71.0%)	987 (75.3%)
	Night with light		661 (16.5%)	242 (18.5%)
	Darkness *		495 (12.5%)	82 (6.2%)
	Non-intersections		Intersections	
GDP (10^8^)	Range	Mean (S.D.)	Range	Mean (S.D.)
	Min: 0.02; Max: 1.10	0.332 (0.284)	Min: 0.02; Max: 1.1	0.352 (0.297)

Note: * represents the variables treated as the control.

**Table 2:** Goodness-of-fit measures for basic and mixed ordered logit models.

Model Statistics	Non-Intersections		Intersections	
	Basic	Mixed	Basic	Mixed
Number of observations, *n*	4005	4005	1311	1311
Number of parameters, *K*	13	18	14	19
Log likelihood at zero, *LL*(**0**)	−4397.18	−4397.18	−1324.15	−1324.15
Log likelihood at convergence, *LL*(**β**)	−3976.79	−3962.27	−1179.59	−1174.91
AIC	7979.6	7960.5	2387.8	2387.2
*Likelihood-ratio test*				
$X^{2}=-2\left[LL(\beta_{random})-LL(\beta_{fixed})\right]$	14.541		4.688	
Degrees of freedom	5		5	
*p*-value	0.01		0.45	

**Table 3:** Estimate results for mixed ordered logit models.

Variables	Non-Intersections			Intersections		
	Mean	Standard Error	z-Statistic	Mean	Standard Error	z-Statistic
Above 60 years old	0.665	0.130	5.115	0.639	0.238	2.685
*s.d. Above 60*				*1.167*	*0.228*	
Without helmets	0.531	0.077	6.896	0.707	0.141	5.014
*s.d. Without helmet*	*0.870*	*0.041*	*21.220*	*0.597*	*0.074*	*8.068*
Equal duty	0.294	0.092	3.196	0.691	0.148	4.669
*s.d. Equal duty*	*0.882*	*0.085*	*10.376*	*0.412*	*0.125*	*3.296*
Three-wheel motorcycle	−1.223	0.101	−12.109	−1.241	0.192	−6.464
*s.d. Three-wheel motorcycle*	*1.103*	*0.094*	*11.734*	*1.440*	*0.186*	*7.742*
Collision vehicles						
Heavy vehicles	0.909	0.090	10.100	1.260	0.172	7.326
*s.d. Heavy vehicles*	*0.683*	*0.082*	*8.329*	*0.796*	*0.161*	*4.944*
National and provincial roads	−0.400	0.086	−4.651			
Road alignment						
Straight				−0.368	0.138	−2.667
Curve and slope	0.329	0.112	2.938			
No Signal control				−0.485	0.131	−3.702
Light conditions						
Daylight	−0.503	0.074	−6.797	−0.962	0.237	−4.059
Night with light				−0.829	0.268	−3.093
*s.d. Night with light*				*0.635*	*0.138*	*4.601*
GDP	0.521	0.124	4.202			

Note: *s.d.* denotes the abbreviation of standard deviation; the italicized represents estimates for the variables resulting in random parameters.

**Table 4:** Estimate results for marginal effects of risk factors.

Variables	Non-Intersections				Intersections			
	PDO	Slight Injury	Serious Injury	Fatality	PDO	Slight Injury	Serious Injury	Fatality
Above 60 years old	−0.574	−0.057	0.454	0.552	−0.601	−0.058	0.402	0.441
Without helmets	−0.618	0.005	0.301	0.315	−0.933	−0.002	0.355	0.344
Equal duty	−0.291	−0.013	0.187	0.208	−0.700	−0.050	0.417	0.445
Three-wheel motorcycle	1.776	−0.105	−0.570	−0.566	2.100	−0.080	−0.507	−0.467
Collision vehicles								
Heavy vehicles	−0.776	−0.082	0.621	0.772	−1.056	−0.157	0.828	1.019
National and provincial roads	0.463	−0.003	−0.227	−0.238				
Road alignment								
Straight					0.403	0.019	−0.211	−0.215
Curve and slope	−0.318	−0165	0.212	0.240				
No signal control					0.590	0.012	−0.258	−0.255
Light conditions								
Daylight	0.496	0.022	−0.320	−0.360	0.942	0.081	−0.592	−0.654
Night with light					1.204	−0.019	−0.386	−0.365
GDP	−0.184	−0.003	0.104	0.112				

Note: PDO denotes the abbreviation of property damage only.

## Abbreviations

The following abbreviations are used in this manuscript:

GDP	Gross Domestic Product
US	United States
UK	United Kingdom
AIC	Akaike Information Criterion
CNY	Chinese Yuan
USD	United States Dollar
